# Challenges constraining availability and affordability of insulin in Bengaluru region (Karnataka, India): evidence from a mixed-methods study

**DOI:** 10.1186/s40545-019-0190-1

**Published:** 2019-10-17

**Authors:** Gautam Satheesh, M. K. Unnikrishnan, Abhishek Sharma

**Affiliations:** 1 Department of Pharmacy Practice, National College of Pharmacy, Kozhikode, Kerala India; 2 Department of Global Health, Boston University School of Public Health, Boston, MA USA; 3 Precision Xtract (Health Economics & Outcomes Research), 101 Tremont Street, Boston, MA 02108 USA

**Keywords:** Access to insulin, Diabetes care, Online pharmacies, India

## Abstract

**Introduction:**

Considering limited global access to affordable insulin, we evaluated insulin access in public and private health sectors in Bengaluru, India.

**Methods:**

Employing modified WHO/HAI methodology, we used mixed-methods analysis to study insulin access and factors influencing insulin supply and demand in Bengaluru in December 2017. We assessed insulin availability, price and affordability in a representative sample of 5 public-sector hospitals, 5 private-sector hospitals and 30 retail pharmacies. We obtained insulin price data from websites of government Jan Aushadhi scheme (JAS) and four online private-sector retail pharmacies. We interviewed wholesalers in April 2018 to understand insulin market dynamics.

**Results:**

Mean availability of insulins on India’s 2015 Essential Medicine List was 66.7% in the public sector, lower than private-sector retail (76.1%) and hospital pharmacies (93.3%). Among private retailers, mean availability was higher among chain (96.7%) than independent pharmacies (68.3%). Non-Indian companies supplied 67.3% products in both sectors. 79.1% products were manufactured in India, of which 60% were marketed by non-Indian companies.

In private retail pharmacies, median consumer prices of human insulin cartridges and pens were 2.5 and 3.6 times, respectively, that of human insulin vials. Analogues depending on delivery device were twice as expensive as human insulin. Human insulin vials were 18.3% less expensive in JAS pharmacies than private retail pharmacies. The lowest paid unskilled worker would pay 1.4 to 9.3 days’ wages for a month’s supply, depending on insulin type and health sector. Wholesaler interviews suggest that challenges constraining patient insulin access include limited market competition, physicians' preference for non-Indian insulins, and the ongoing transition from human to analogue insulin. Rising popularity of online and chain pharmacies may influence insulin access.

**Conclusion:**

Insulin availability in Bengaluru’s public sector falls short of WHO’s 80% target. Insulin remains unaffordable in both private and public sectors. To improve insulin availability and affordability, government should streamline insulin procurement and supply chains at different levels, mandate biosimilar prescribing, educate physicians to pursue evidence-based prescribing, and empower pharmacists with brand substitution. Patients must be encouraged to shop around for lower prices from subsidized schemes like JAS. While non-Indian companies dominate Bengaluru’s insulin market, rising market competition from Indian companies may improve access.

**Electronic supplementary material:**

The online version of this article (10.1186/s40545-019-0190-1) contains supplementary material, which is available to authorized users.

## Introduction

The increasing global burden of non-communicable diseases (NCDs) poses a major public health challenge. The 2030 Agenda for Sustainable Development has prioritized the reduction of premature mortality due to NCDs by a third [1]. Diabetes is one of the most prevalent NCDs and its complications severely affect patients’ quality of life, finances as well as the national economy [2]. Insulin is an essential, life-saving medicine for treating type 1 and 2 diabetes. World Health Organization (WHO) identifies Essential Medicines as those which meet the global health needs of the majority population and promote cost-effective use of healthcare resources [3]. Nearly 100 years since its discovery, insulin remains inaccessible to millions due to poor availability and unaffordable prices [4–6]. Therefore, various global health institutions have called for assessment of insulin availability and affordability in local contexts [4, 7–9].

India is second only to China in terms of diabetes burden. Among leading causes of death, diabetes was associated with the highest increase (⁓ 63%) in age-adjusted mortality during 1990–2016 [10]. In India, some insulin products are listed on the national and state essential medicines lists (EMLs) for free-of-charge provision in the public-sector health facilities [11]. However, in general, India’s central and state public health systems are under-funded and offer limited healthcare coverage to the majority of the population [12]. This forces diabetes patients to seek healthcare in the private sector through out-of-pocket (OOP) payments [13]. Patients also purchase medicines through private-sector online pharmacies and/or government schemes such as Jan Aushadhi Scheme (JAS) that aim to provide quality medicines at affordable prices to all [14–16].

In this context, it is important to evaluate insulin availability and affordability in India’s healthcare sector. A previous study on insulin access in the northern state of Delhi was limited to the private sector market [16]. Further, the different states in India (often described as ‘nations within nation’) have stark differences in disease burden and health system functioning, which calls for region-specific studies [10]. Presented here is the first study evaluating challenges constraining insulin access in both public and private health sectors as well as the factors influencing insulin uptake and demand in Bengaluru (formerly, Bangalore) region in India’s southern state Karnataka.

## Methods

We conducted a mixed methods analysis to study insulin availability, prices, and factors influencing insulin supply and demand (market dynamics) in both public and private health sectors of Bengaluru region. We employed a modified version of the WHO/Health Action International (WHO/HAI) survey to assess insulin availability and prices. While a standard WHO/HAI survey collects availability and price data on pre-defined list of essential medicines, we surveyed all the insulin products available at the survey facilities. We also obtained insulin price data from the websites of various online pharmacies supplying insulin in Bengaluru (see Additional file 1: Table S1 for website references). We conducted an insulin affordability analysis, in line with WHO/HAI methodology.

To understand the market dynamics, we conducted in-depth interviews with wholesalers in the private-sector market and conducted qualitative thematic analyses using inductive approach. We developed the interview guide based on the published literature on medicines access in India, our prior work on insulin access [5, 17, 18], and observations made during the facility survey around the factors influencing insulin access in the region. See Additional file 1 for interview guide. We identified major insulin wholesalers who supplied insulin to several of the surveyed retail pharmacies in Bengaluru’s private-sector market. We then conducted in-depth qualitative interviews with these experienced wholesalers until we achieved response saturation.

### Sampling

Bengaluru – Karnataka state’s capital and India’s information technology and biotechnology hub – is a highly populated urban region with a high diabetes burden. Bengaluru is divided into five administrative zones, namely Bengaluru North, Bengaluru South, Bengaluru East, Bengaluru West and Bengaluru Central. In addition, Bengaluru further expands to peripheral towns. We conducted a representative facility survey – in four randomly selected zones and an additional peripheral town – to collect insulin price data in December 2017, and qualitative data from interviews in April 2018.

#### Survey medicines

Insulin is available from various sources (animal, human and analogues), in different strengths (40, 100 IU/mL) and delivery devices (vials, pens, cartridges). In this survey, we define an “insulin product” as a unique combination of any insulin molecule, strength, presentation (delivery device) and manufacturer/company. Considering variations in marketed insulin products, we acquired price data for all the unique insulin products available at a given pharmacy surveyed.

#### Survey facilities

Out of five zones mentioned above, we randomly selected four survey zones and one peripheral town (fifth zone). In each survey zone, we selected one public-sector secondary or tertiary level hospital as the “survey anchor” and one private-sector hospital (i.e. total 10 hospitals were surveyed). The survey anchor facilities are the major/largest public-sector hospitals, in terms of bed count and facility for out-patient/primary care services. See Additional file 1: Table S1 for details. Since there exists no publicly-available list of private pharmacies, we surveyed private-sector retail pharmacies – in randomly selected direction(s) – within 2 Kms from the survey anchor facility. This involved data collectors to stand outside each public-sector hospital entrance (survey anchor) – as a caregiver or a patient would – and pick up a random direction/road to purchase insulin from private-sector pharmacy. We surveyed six private-sector pharmacies in each of five survey zones (*n* = 30 private-sector pharmacies: 10 chain pharmacies and 20 independent pharmacies).

Our study also included four online pharmacies selected using convenience sampling. Price data were collected from websites of these online pharmacies, primarily for the insulin products that we found in our facility survey.

### Data collection and analysis

Upon explaining the purpose of our survey to the pharmacy personnel, we acquired data on consumer prices of all the unique insulin products available at the survey facilities. We inquired the pharmacists about any insulin discounts they offered along with other problems in access, and obtained the addresses of wholesalers supplying insulin products.

From the product label, we classified the insulin products based on the country where corporate headquarters of the company is located as well as the country where the product was manufactured. In other words, we identified if the insulin was ‘imported’ into India for sale by an Indian or Non-Indian company or if it was made in India (domestically manufactured) for sale by an Indian or Non-Indian company. This information on country of manufacture (i.e., India or Outside India) and company corporate headquarters (Indian or Non-Indian) results in four combinations (See Table 1).

**Table 1:** Distribution of insulin products in Bengaluru region, by insulin type, place of manufacture and marketing companies

a. Private-sector retail (*N* = 30) and hospital pharmacies (*N* = 5)			
		Products Marketed by Indian companies (Biocon, Wockhardt, Lupin, Cadila, Nicholas Piramal, Ranbaxy)	Products Marketed by Non-Indian Companies (Novo Nordisk, Eli Lilly and Sanofi)
Place of Manufacture	INDIA	Total (*N* = 109) Human = 89.0% (*n* = 97) Analogue = 10.1% (*n* = 11) Porcine = 0.9% (*n* = 1)	Total (*N* = 159) Human = 81.1% (*n* = 129) Analogue = 18.2% (*n* = 29) Porcine = 0.6% (*n* = 1)
	OUTSIDE INDIA	Total (*N* = 0)	Total (*N* = 72) Huma*n* = 40.3% (*n* = 29) Analogue = 59.7% (*n* = 43)
b. Public-sector hospital pharmacies (*n* = 5)			
		Products Marketed by Indian companies (Biocon, Lupin, Cadila Healthcare)	Products Marketed by Non-Indian Companies (Novo Nordisk, Eli Lilly and Sanofi)
Place of Manufacture	INDIA	Total (*N* = 11) Human = 81.8% (*n* = 9) Analogue = 18.2% (*n* = 2)	Total (*N* = 11) Human = 90.9% (*n* = 10) Analogue = 9.1% (*n* = 1)
	OUTSIDE INDIA	Total (*N* = 0)	Total (*N* = 6) Human = 33.3% (*n* = 2) Analogue = 66.7% (*n* = 4)

‘n’ indicates total number of products included in a given segment, irrespective of dosage, strength and delivery device

We calculated the mean availability of insulin products – stratified by insulin type, strength and delivery device – as percentage of facilities where a given insulin was found. Mean availability is reported for both the public (i.e. government hospitals) and private (retail and hospital pharmacies) sectors. For the private retail pharmacies, we compared the availability at chain and independent pharmacies. For both public and private sectors, we calculated ‘brand occurrence’ i.e. the proportion of surveyed facilities that stocked a given insulin product (brand) from a specific company.

For price analyses, we also calculated the median prices of all the human and analogue insulin products found in survey facilities as well as online pharmacies, adjusted to an internal standard of 100 IU/10 mL to facilitate meaningful comparisons. We assessed the statistical significance of price differences (α significance level of 0.05), by non-parametric Wilcoxon rank-sum tests using statistical software SAS Version 9.4. The affordability was estimated as per the WHO/HAI methodology; i.e. insulin is considered unaffordable if the lowest paid unskilled worker has to work for more than a day to afford insulin supply for 1 month. Additionally, we conducted thematic analysis of the notes taken during the qualitative interviews of experienced insulin wholesalers. Two independent reviewers assessed the wholesaler interview scripts to identify major themes and interpret the findings.

## Results

We found a total of 368 individual insulin products (including duplicates i.e. same insulin molecule – of same strength/delivery device - manufactured and marketed by different companies) across the 40 pharmacies surveyed in Bengaluru’s public and private sector combined. Most surveyed facilities had stocked insulin sold by both Indian and Non-Indian companies. In the private sector, 75.0 and 24.4% of all the 340 products found (including duplicate products) were human and analogue insulins, respectively. Additionally, porcine insulin constituted 0.6% (*n* = 2) of the products delivered in just one private-sector hospital run by a charitable trust. Majority of the products were manufactured in India (268/340, i.e. 78.8%). 86.2% (*n* = 231) of the products manufactured in India were supplied to Non-Indian companies (for instance: Huminsulin® was manufactured in India for Eli Lilly, a Non-Indian company). Only 21.1% of insulin products were imported, all of which were marketed by Non-Indian companies.

In the five surveyed public-sector facilities, we found a total of 28 insulin products (analogue insulins: 25.0%; human insulin: 75.0%, including duplicate products). About 39.0% (*n* = 11) of these products were from Indian companies i.e. Biocon, Cadilla Healthcare, Nicholas Piramal, Ranbaxy, Wockhardt and Lupin. In the private-sector retail and hospital pharmacies, 75% of total 340 products were human insulin. See Table 1. In both public and private sectors, Actrapid® 40 IU/ml, was the most widely available brand for soluble insulin (Additional file 1: Tables S2-S4).

### Facility survey: insulin availability

The surveyed facilities in both public (5 hospitals) and private sector (30 retail pharmacies and 5 hospitals), in general, stocked four types of human insulins in 40 and 100 IU/ml strength, in addition to seven types of analogues, all in 100 IU/ml strength. Both human and analogue insulins were available in vial, cartridge and pen delivery devices. Additionally, one type of porcine insulin (40 IU/ml vial) was found in one private-sector charitable hospital.

Table 2 presents availability of various insulin products. The mean availability of insulin products (on India’s 2015 EML) was 66.7% in the public-sector hospitals as compared to 76.1 and 93.3%, in the private-sector retail and hospital pharmacies respectively [14]. Soluble insulin and biphasic isophane insulin 30/70 in 40 IU/ml strength were available in 80% of surveyed facilities in the public sector. Generally, a wider range of insulin products was available in the private sector than in the public sector. Only a few public-sector pharmacies stocked insulin analogues.

**Table 2:** Availability of insulin products in the private and public sectors in Bengaluru region

Insulin type	Molecule description	Strength	Percentage availability		
			Private-sector		Public-sector
			Retail pharmacies [*N* = 30]	Hospital pharmacies [*N* = 5]	Hospital pharmacies [*N* = 5]
Human insulin	Soluble Insulin	40 IU/ml^a^	83.3% (*n* = 25)	80.0% (*n* = 4)	80.0% (*n* = 4)
		100 IU/ml	33.3% (*n* = 10)	80.0% (*n* = 4)	20.0% (*n* = 1)
	Isophane Insulin	40 IU/ml^a^	51.5% (*n* = 17)	100.0% (*n* = 5)	40.0% (*n* = 2)
		100 IU/ml	16.7% (*n* = 5)	20.0% (*n* = 1)	0.0%
	Biphasic Isophane Insulin: 30% Soluble 70% Isophane Insulin	40 IU/ml^a^	93.3% (*n* = 28)	100.0% (*n* = 5)	80.0% (*n* = 4)
		100 IU/ml	66.7% (*n* = 20)	80.0% (*n* = 4)	40.0% (*n* = 2)
	Biphasic Isophane Insulin: 50% Soluble 50% Isophane Insulin	40 IU/ml	20.0% (*n* = 6)	40.0% (*n* = 2)	20.0% (*n* = 1)
		100 IU/ml	3.3% (*n* = 1)	0.0%	0.0%
Analogue insulin	Insulin Aspart	100 IU/ml	33.3% (*n* = 10)	60.0% (*n* = 3)	20.0% (*n* = 1)
	Biphasic insulin aspart: 30% insulin aspart & 70% protamine crystallized aspart	100 IU/ml	36.7% (*n* = 11)	20.0% (*n* = 1)	20.0% (*n* = 1)
	Insulin glargine	100 IU/ml	66.7% (*n* = 20)	60.0% (*n* = 3)	60.0% (*n* = 3)
	Insulin glulisine	100 IU/ml	0.0%	0.0%	0.0%
	Insulin detemir	100 IU/ml	3.3% (*n* = 1)	20.0% (*n* = 1)	0.0%
	Insulin lispro	100 IU/ml	6.7% (*n* = 2)	20.0% (*n* = 1)	0.0%
	Insulin lispro biphasic: 25% insulin lispro 75% lispro protamine	100 IU/ml	13.3% (*n* = 4)	0.0%	0.0%
	Insulin lispro biphasic: 50% insulin lispro 50% lispro protamine	100 IU/ml	20.0% (*n* = 6)	60.0% (*n* = 3)	20.0% (*n* = 1)
Animal Insulin	Porcine soluble insulin	40 IU/ml	–	20.0% (*n* = 1)	–
	Porcine soluble insulin	100 IU/ml	–	–	–
Mean availability of human insulins listed in 2015 India’s essential medicines list			76.1%	93.3%	66.7%

^a^Insulins listed on 2015 India’s essential medicines list

Retail pharmacies more often stocked human insulin products marketed by non-Indian companies than by Indian companies (96.7% vs 73.3%). Biocon was the only Indian company that marketed analogue insulin. Majority (80%) of the retail pharmacies stocked at least one of the insulin analogues. 26.7 and 76.7% facilities had analogue insulins by Indian and non-Indian companies, respectively. See Table 3. Within the private retail sector, mean insulin availability was higher among chain pharmacies (96.7%) compared to independent pharmacies (68.3%). See Additional file 1: Table S5.

**Table 3:** Availability of insulin in Bengaluru’s private-sector retail pharmacies based on type, delivery device and company type

a. Private-sector retail pharmacies [*N* = 30]			
Insulin type and delivery device	Company Type		
	Indian	Non-Indian	Combined (Indian & Non-Indian)
Human insulin (overall)	73.3% (*n* = 22)	96.7% (*n* = 29)	96.7% (*n* = 29)
Vial			
40 IU/ml	66.7% (*n* = 20)	96.7% (*n* = 29)	96.7% (*n* = 29)
100 IU/ml	50.0% (*n* = 15)	43.3% (*n* = 13)	66.7% (*n* = 20)
Cartridge			
40 IU/ml	0.0% (*n* = 0)	0.0% (*n* = 0)	0.0% (*n* = 0)
100 IU/ml	0.0% (*n* = 0)	30.0% (*n* = 9)	30.0% (*n* = 9)
Pen			
40 IU/ml	0.0% (*n* = 0)	0.0% (*n* = 0)	0.0% (*n* = 0)
100 IU/ml	0.0% (*n* = 0)	36.6% (*n* = 11)	36.7% (*n* = 11)
Analogue insulin (overall)	26.7% (*n* = 8)	76.7% (*n* = 23)	80.0% (*n* = 24)
Vial			
40 IU/ml	0.0% (*n* = 0)	0.0% (*n* = 0)	0.0% (*n* = 0)
100 IU/ml	20.0% (*n* = 6)	0.0% (*n* = 0)	20.0% (*n* = 6)
Cartridge			
40 IU/ml	0.0% (*n* = 0)	0.0% (*n* = 0)	0.0% (*n* = 0)
100 IU/ml	10.0% (*n* = 3)	30.0% (*n* = 9)	33.3% (*n* = 10)
Pen			
40 IU/ml	0.0% (*n* = 0)	0.0% (*n* = 0)	0.0% (*n* = 0)
100 IU/ml	0.0% (*n* = 0)	73.3% (*n* = 22)	73.3% (*n* = 22)
b. Public-sector hospital pharmacies [*N* = 5]			
Human insulin (overall)	80.0% (*n* = 4)	60.0% (*n* = 3)	80.0% (*n* = 4)
Vial			
40 IU/ml	80.0% (*n* = 4)	60.0% (*n* = 3)	80.0% (*n* = 4)
100 IU/ml	60.0% (*n* = 3)	20.0% (*n* = 1)	60.0% (*n* = 3)
Cartridge			
100 IU/ml	0.0% (*n* = 0)	20.0% (*n* = 1)	20.0% (*n* = 1)
Pen			
100 IU/ml	0.0% (*n* = 0)	20.0% (*n* = 1)	20.0% (*n* = 1)
Analogue insulin (overall)	40.0% (*n* = 2)	40.0% (*n* = 2)	60.0% (*n* = 3)
Vial			
40 IU/ml	0.0% (*n* = 0)	0.0% (*n* = 0)	0.0% (*n* = 0)
100 IU/ml	40.0% (*n* = 2)	0.0% (*n* = 0)	40.0% (*n* = 2)
Cartridge			
100 IU/ml	0.0% (*n* = 0)	20.0% (*n* = 1)	20.0% (*n* = 1)
Pen			
100 IU/ml	0.0% (*n* = 0)	40.0% (*n* = 2)	40.0% (*n* = 2)

### Facility survey: insulin prices and affordability

Table 4 summarizes the consumer prices of human and analogue insulin (adjusted to 100 IU/ml 10 ml pack) in the private-sector retail pharmacies by place of manufacture (‘in India’ or ‘outside India’) and by company’s origin (Indian or non-Indian). The median prices of human insulin cartridges and pens were 2.5 and 3.6 times higher than the price in vial form (USD 4.97 | INR 323.5) respectively. Median consumer prices of analogue insulins were higher than that of human insulins irrespective of dosage form. Price of analogue insulin vial (USD 17.96), cartridge (USD 27.56) and pen (USD 33.20) were 3.6, 5.5 and 6.7 times higher than that of human insulin vials (USD 4.97). Prices of human insulin vials and analogue insulin cartridges marketed by Indian companies were 5.5 and 18.5% respectively, cheaper than those by non-Indian companies. Human insulin vials of 40 IU/ml strength were the least expensive. Insulin prices were similar in the public-sector hospital pharmacies (see Additional file 1: Table S6b). The lowest paid unskilled government employee in Bengaluru has to work 1.4 days and 9.3 days, respectively, to purchase monthly supply of insulin depending on insulin type, delivery device and sector. Furthermore, our estimated per capita monthly cost of insulin ranged from over 0.9 to about 9.2 times India’s per-capita monthly health spending in the public and private sectors (See Additional file 1: Table S6).

**Table 4:** Median consumer prices (adjusted to 10 ml 100 IU/ml) in Bengaluru’s private retail pharmacies, by insulin type, place of manufacture and company’s origin

a. By place of manufacture				
	Combined (imported + domestic)	Manufactured outside India	Manufactured in India	*p*-value
Human insulin				
Vial	USD 4.97 | INR 323.5 (*n* = 187)	--	USD 4.97 | INR 323.5 (*n* = 187)	
Cartridge	USD 12.24 | INR 796.6 (*n* = 9)	USD 12.24 | INR 796.6 (*n* = 7)	USD 13.55 | INR 881.6 (*n* = 2)	0.0278^a^
Pen	USD 17.98 | INR 1170.0 (*n* = 16)	USD 17.98 | INR 1170.0 (*n* = 16)	-- --	
Analogue insulins				
Vial	USD 17.96 | INR 1168.8 (*n* = 6)	–	USD 17.96 | INR 1168.8 (*n* = 6)	
Cartridge	USD 27.56 | INR 1794.0 (*n* = 12)	–	USD 27.56 | INR 1794.0 (*n* = 12)	
Pen	USD 33.20 | INR 2160.0 (*n* = 49)	USD 32.47 | INR 2113.3 (*n* = 33)	USD 42.66 | INR 2775.6 (*n* = 16)	< 0.0001^a^
b. By company’s origin				
	Combined (Non-Indian + Indian)	Non-Indian Company	Indian Company	
Human insulin				
Vial	USD 4.97 | INR 323.5 (*n* = 187)	USD 5.15 | INR 335.2 (*n* = 105)	USD 4.86 | INR 316.7 (*n* = 82)	< 0.0001^a^
Cartridge	USD 12.24 | INR 796.6 (*n* = 9)	USD 12.24 | INR 796.6 (*n* = 9)	–	
Pen	USD 17.98 | INR 1170.0 (*n* = 16)	USD 17.98 | INR 1170.0 (*n* = 16)	–	
Analogue insulin				
Vial	USD 17.96 | INR 1168.8 (*n* = 6)	–	USD 17.96 | INR 1168.8 (*n* = 6)	
Cartridge	USD 27.56 | INR 1794.0 (*n* = 12)	USD 28.60 | INR 1861.3 (*n* = 18)	USD 23.32 | INR 1517.6 (*n* = 3)	0.0142^a^
Pen	USD 33.20 | INR 2160.0 (*n* = 49)	USD 33.20 | INR 2160.0 (*n* = 49)	–	

^a^The difference in median prices in the groups (1. Manufactured outside (imported) versus in India (domestic), and 2. Company’s origin being non-Indian versus Indian) are statistically significant at α-significance level of 0.05 (Wilcoxon rank sum test)

### Online pharmacy and Jan Aushadhi scheme

Some insulin products – such as human insulin vials and cartridges – were less expensive at online pharmacies than private retail pharmacies, while others were not. See Table 5. Compared to retail pharmacies, we found a larger variety of insulin products and brands in the surveyed online pharmacies. Human insulin vials were 8.3% less expensive in JAS pharmacies (USD 4.55 | INR 296.5) compared to private retail pharmacies.

**Table 5:** Median consumer prices of insulin: online pharmacies Vs. retail pharmacies – adjusted to 100 IU 10 ml pack

	Online pharmacy prices (INR)					Retail pharmacy prices (INR)	p-value (retail vs overall online pharmacy)
	1 mg	Apollo	MedPlus	NetMeds	Overall (i.e. all online pharmacies combined)		
Human insulin							
Vial	301.0	322.2	289.9	322.2	321.8 (*n* = 93)	323.5 (*n* = 187)	< 0.0001^a^
Cartridge	794.8	585.5	715.5	590.2	654.8 (*n* = 40)	796.6 (*n* = 9)	0.2630
Pen	1092.7	1213.3	1092.0	1213.3	1213.3 (*n* = 9)	1170.0 (*n* = 16)	0.0545
Insulin analogues							
Vial	1474.7	1421.8	1253.5	1633.3	1575.3 (*n* = 13)	1168.8 (*n* = 6)	0.0653
Cartridge	1920.0	1703.6	1578.1	1662.3	1672.2 (*n* = 33)	1794.0 (*n* = 12)	0.7273
Pen	2190.3	2240.0	2016.0	2141.1	2190.2 (*n* = 33)	2160.0 (*n* = 49)	0.0181^a^

^a^The difference in median prices in retail pharmacy facilities and online pharmacies are statistically significant at α-significance level of 0.05 (Wilcoxon rank sum test) 'n' refers to the number of price data points used to calculate median consumer prices

## Qualitative results

Of the six identified major wholesalers, four (66.6%) participated in our in-depth interviews that lasted for 28–55 min. These wholesalers have been supplying insulin to at least 10 pharmacy facilities in Bengaluru region for over a decade.

Three major themes emerged from interviews with wholesalers. The thematic factors influencing insulin uptake and demand (market dynamics) included (1). limited market competition, (2). ongoing transition from human insulin to analogues, and (4). insulin supply by different pharmacy types. Wholesalers’ views were based on their own experiences and business practices.

### Limited market competition

Responding to our observation that most surveyed pharmacies stocked insulin by Non-Indian companies, the wholesalers said that insulin uptake is largely driven by physician prescribing. Physicians continue to prefer insulin products marketed by Non-Indian companies over their Indian counterparts, even though the latter is often less expensive. Most patients adhere to the insulin brands prescribed by physicians, resulting in a persistent demand for insulin products marketed by Non-Indian companies.

“*Our supply of insulin products to retailers is based on what products customers demand the most, which traces back to what the doctors prescribe*.” - Wholesaler A

While insulin market in Bengaluru appears to be oligopolistic, many pharmacies we surveyed had insulin products, including analogue insulin, marketed by Indian companies such as Biocon. Wholesalers said that the uptake of Indian company’s insulin is increasing but it remains insignificant in comparison to the Non-Indian companies’ by volume of market share. Wholesalers added that Indian companies would need intense marketing strategies to increase their market share. Biocon, headquartered in Bengaluru, has a regional advantage.

“*The reason for Biocon’s growth could be attributed to its considerable investment in R&D. They now manufacture anticancer drugs, and this has indirectly popularized even their insulin products among doctors, especially in Bangalore. Yes, there is a regional advantage. Although Biocon has been growing steadily over the last 10-15 years, Novo Nordisk continues to be the pioneer in the insulin market*.” - Wholesaler B

Furthermore, wholesalers find it unprofitable to stock insulin products by certain Indian companies owing to inadequate demand and the market dominance by Non-Indian companies. Indian companies like Ranbaxy and Wockhardt, which manufacture and market multiple medicines, do not adequately market or popularize their insulin products. Ultimately, both wholesalers and retailers stock insulin products (i.e. primarily those by Non-Indian companies) which help them make profits.

*“The wholesaler prices depends on the quantity of order, regardless of hospital or chain pharmacies. The maximum discount can go up to 20%. However, we do not sell insulin directly to customers. We supply to retailers only.” - Wholesaler C.*

### Transition from human insulin to analogues

In our survey, analogue insulins – mostly found in the pen and cartridge form - were available in both private-sector hospitals and retail pharmacies. Wholesalers said that expensive analogue pens/cartridges are now available more in the private sector, at least in part, because patients find those easy to administer. However, this is not true in rural Bengaluru. Less than half of the retail pharmacies in rural Bengaluru stocked analogues. Wholesalers reported that the poor sales of analogues in the rural areas could solely be attributed to its unaffordability.

“*Analogue pens are becoming popular because most of the customers in urban Bengaluru do not mind paying for the expensive pens. Many patients prefer single dosing using a pen than repeated dosing with insulin syringe. They choose convenience over cost*. *But this is not the case in rural Bengaluru, where people can hardly afford the human insulin vials*.” - Wholesaler B.

### Insulin supply by different pharmacy types

In our facility survey, we found that chain pharmacies had slightly higher availability of insulin compared to the independent pharmacies. Wholesalers reported that a chain pharmacy can arrange the delivery of an out-of-stock medicine within a few hours. However, this would be a difficult task for most independent pharmacies because they order insulin on a weekly or monthly basis. Furthermore, two of the leading chain pharmacies in the region, in addition to hundreds of their retail units, also run online pharmacies; thereby increasing their popularity. All of this helps in establishing patients’ trust in the pharmacy chain, ensuring repeat (regular) customers.

“*From a wholesaler’s point of view, both chain pharmacies and independent units are somewhat similar. However, when customers see multiple branches under the same name all around the city, there is a notion that the chain pharmacy is good*. *We supply the retailers based on the [purchase] order they place, and not based on the number of retail outlets they have established in the city. However, chain pharmacies often have substantially higher sales than standalone units, and therefore, we are obliged to supply products to them as frequently*.” - Wholesaler D

Wholesalers said that many patients in Bengaluru are now buying their medications from online pharmacies, owing to heavy concessions and quick door delivery. Although online pharmacies ensure timely delivery of almost all prescription and over-the-counter medicines, one must reconsider purchasing insulin online. Wholesalers said that almost all damaged goods in an online purchase could arguably be replaced in a certain period of time. However, there is no room for trial and error when it comes to essential, life-saving medicines like insulin. Inappropriate storage and transportation conditions can render insulin inactive and devoid of clinical utility.

“*Through online pharmacies, patients in Bengaluru enjoy door delivery of medicines, at attractive prices. But online purchasing of insulin presents a different case. Many online customers of insulin frequently report delivery of broken vials and damaged pens. Moreover, the storage conditions are questionable, unlike in retail pharmacies*. *I agree that the damaged products are easily replaceable, but no patient should be put to wait for a lifesaving medicine like insulin*.” - Wholesaler B

## Discussion

To our best knowledge, this is the first study to assess insulin availability, prices and access issues in both the public and private health sectors in a Southern state in India. Existing literature notes that insulin remains inaccessible to patients globally, particularly in low and middle income countries, owing to poor availability, limited market competition, and unaffordable prices [4, 6, 17, 19]. Reports suggest that mean private-sector insulin availability in 15 counties was as low as 39.0% (range: 0.0% in Kyrgyzstan to 95.0% in Kwazulu-Natal State, South Africa), with only two countries South Africa [2011] and Lebanon [2013]) having met WHO’s target of at least 80% availability [4]. Other studies report that private-sector availability of human insulin was 44.4% in Delhi state (India) and 20.0% in Kathmandu Valley (Nepal) [5, 17]. In Hubei province (China), insulin availability was higher in public-sector hospitals (70–90%) than in private-sector retail pharmacies (13–33%) [6].

In Bengaluru (Karnataka state), the public-sector availability (66.7%) of human insulins listed on 2015 India’s EML was below WHO’s 80% target. In contrast, private-sector hospitals and retail pharmacies (especially chain pharmacies) performed better in availability. For analogue insulin, the availability was higher in the private-sector retail pharmacies than in the public sector. This may be because the public-sector medicine formularies/procurement are influenced by the local EMLs, and the Karnataka EML does not list any analogue insulins. See Table 2 and Additional file 1: Table S5.

Although Karnataka – like some other Indian states - has its own EML, we included insulin listed on India’s 2015 EML (a guiding document for state-specific EMLs) for overall availability calculation. The reason being: the 2014–2015 Karnataka’s EML mentions one of the two listed insulins by brand name i.e. Actrapid® (soluble human insulin 40 IU/ml marketed by Non-Indian company Novo Nordisk) [20]. We expect such practices – i.e. mentioning brand instead of generic/molecule name – would restrict insulin market competition and adversely impact clinical practice, defeating the *raison d’être* of EML. This might be a reason why Actrapid® was available in as many as 83% of surveyed facilities in the private sector and 70% in the public sector facilities (see Additional file 1: Tables S2-S3).

It is surprising that this glaring anomaly has not caught the attention of the pharmaceutical regulatory authorities over the past 3 years, indicating the dismal quality of review and discussion on such critically important issues. In 2002, Karnataka Drug Logistics & Warehousing Society (KDLWS) was established for selection and procurement of essential medicines, and for the supply of those essential medicines free-of-cost to the public health facilities. A review of KDLWS stocks in July 2013 revealed “a significant number of stock-outs on that day. Overall, 24.0% of items (medicines) were out of stock in 80.0% or more of the warehouses and only 23.0%…were available in all warehouses” [21]. Such a situation apparently holds true even today; our survey has demonstrated the limited insulin availability in public-sector hospital pharmacies. Contrary to KDLWS’ mission and the general belief that government facilities provide essential medicines free-of-cost, we found that all public-sector hospital pharmacies charge prices that are similar to what patients pay for insulin in private sector (see Additional file 1: Table S6).

It appears that the Bengaluru insulin market – like those in Delhi (India) and other emerging countries – is witnessing a transition from human insulin to expensive analogue insulins and cartridges/pen forms of delivery [4, 5, 16, 22]. Recent studies report that, in India, as high as 38.0 and 66.0% patients, respectively, use analogue insulin and insulin pens [23, 24]. However, Baruah et al. – in a cross-sectional registry-based retrospective study in India - found no statistically significant association between hypoglycaemic control and type of insulin [24].

We note that there is an ongoing debate on the clinical effectiveness of insulin analogues that are more expensive and add to the financial burden for health systems and individuals [25, 26]. Evidence – largely industry funded and from higher income countries – indicates that analogue insulin help reduce hypoglycaemic events and weight gain, improve treatment adherence, reduce fear of dose adjustment, and improved patient satisfaction [25, 27]. On the contrary, in a review of 64 comparison studies, only 23% studies showed that analogues were significantly better in lowering A1C levels [26]. This is in line with WHO’s 17th Expert Committee on the Selection and Use of Essential Medicines (2009), which concluded that “insulin analogues currently offer no significant clinical advantage over recombinant human insulin and there is still a concern about possible long-term adverse effects” [28]. In recent proceedings of the WHO’s 22nd Expert Committee (2019), researchers argued that there is no independent evidence from low and middle income countries to support that long-acting analogues are a cost-effective alternative to human insulin. Furthermore, given the oligopolistic global insulin market [29], wider adoption of analogues “could be counterproductive [in global context] and lead to the disappearance of human insulin from the market, as happened with animal insulin” [27, 30].

The global insulin market - including that in Bengaluru - is dominated by few Non-Indian companies, allowing them to shape the markets with substantial increase in uptake of higher priced pens and analogue insulins [4, 5, 17]. Our qualitative findings suggest that this transition is driven by high-income/urban patients’ ability to pay, perceived benefits (such as ease of administration) of analogues, supply chain profits, and prescribing habits of physicians, shaped by marketing forces. Such a transition will have major cost implications for local healthcare system, because analogue insulin pens/cartridges are 5.5–6.7 times more expensive than human insulin vials (Table 4). We note that the lowest paid unskilled worker in Bengaluru would pay 1.4 to 9.3 days’ wages to purchase monthly supply, depending on the insulin type and healthcare sector. Furthermore, patients with diabetes pay even more to treat comorbidities. More importantly, per-patient cost of monthly insulin supply in public and private pharmacy sectors can be as high as six times India’s monthly per capita health spending (Additional file 1: Table S6).

Our analyses indicate that online pharmacies and JAS pharmacies offer insulin at relatively lower prices. Prices at JAS pharmacy – which sells human insulin vials only - were nearly 18% cheaper than private retail pharmacies. See Table 5. However, existing literatures indicate that JAS pharmacies have not achieved much popularity because of government’s inadequate support and poor campaigning, physicians prescribing brands and not generics, poor supply chain, and patient concerns about medicine quality [31, 32]. In this regard, India’s central and state governments should optimize JAS’s medicine list and supply chain, inform patients about the added value of obtaining medicines from JAS, mandate physicians to prescribe generics, and encourage patients to avail discounted insulin prices [33–36]. While online pharmacies could improve insulin access and affordability in India, this not-so-well-regulated sector has implications for insulin quality and supply chain security (see qualitative results) [36, 37]. We recommend comprehensive evaluation and regulation of online pharmacies to harness the potential benefits.

Increasing market competition – particularly from Indian companies – could help improve insulin access and affordability. This could be true in the light of a recent systematic literature review concluding that biosimilar insulin has comparable safety and efficacy to their reference products [38]. Although Biocon has been successful in obtaining regulatory approval for its biosimilar insulin glargine in many foreign countries (European Union, Australia and Japan) [39, 40], physicians in Bengaluru – just as in Delhi (India) - appear to put more trust in insulin by Non-Indian companies. Furthermore, the recently introduced Biocon’s glargine analogue biosimilar is expected to increase market competition. However, this may push non-Indian companies (such as Novo Nordisk, Eli Lilly) to intensify marketing their analogues which face less competition from Biocon. In this regard, educational campaigns focusing on both patients and prescribers are needed to sensitize physicians to patients’ affordability of analogues. Realizing their corporate-social responsibility, Indian insulin manufacturers should collaborate with the government to communicate public health benefits of less-expensive human insulin, and biosimilars whenever analogues are clinically indicated. Physicians should also explore sustainable means to make insulin accessible and affordable to local populations.

India’s central and state governments, in collaboration with healthcare providers and patient organizations, should develop evidence-based guidelines that prioritize use of less expensive, quality-assured human insulin and restrict the use of analogues.. The ongoing policy discussion around empowering pharmacists in India to substitute brands/generics might be a step forward [34, 41]. All this would facilitate healthy market competition among products. The identified insulin access and affordability issues, if ignored, will further worsen the catastrophic public health crisis from diabetes.

## Limitations of the study

Despite the strengths of WHO/HAI methodology, there are some limitations. Our analysis is based on data collected on the day of survey and may not indicate availability and prices over time. Affordability may be overestimated as a large proportion of the population earns less than the lowest wage of an unskilled worker set by the government. Furthermore, the results of our analysis in Karnataka’s capital and urbanized region, Bengaluru, may not be representative of other regions in Karnataka state. However, we would expect our results to be the ‘best-case scenario’, as interviewed wholesalers and previous studies from India have noted lower medicine availability in rural areas [33, 42, 43].

## Conclusion

This study particularly highlights the limited insulin availability in the public-sector hospitals. Insulin remains unaffordable in both private and public sectors. Our qualitative findings suggest that challenges constraining patient insulin access (availability and affordability) include limited market competition in Bengaluru insulin market, physician’s preference for non-Indian insulin products, perceived benefits of insulin analogues and the ongoing transition from human to analogue insulin. While Non-Indian companies dominate Bengaluru’s insulin market, the rising market competition from Indian companies may improve access. This requires proactive policy interventions from the Indian government along with reciprocal approaches from healthcare and patient organizations. Government should mandate generic prescribing, educate physicians to pursue evidence-based insulin initiation and treatment, and empower pharmacists with brand substitution. Streamlining insulin procurement and supply chains (especially in public sector) would improve insulin price transparency and encourage patients to shop around or avail governmental concessions (Jan Aushadhi), thereby improving access. The government must also consider a wider range of insulin procurement options, increase its bargaining power to negotiate for competitive prices, and develop strategies for equitable access to quality-assured insulin.

## Additional file

Additional file 1: Supplementary details on study methods, data analyses, and insulin availability and affordability. (PDF 457 kb)

## Data Availability

The datasets used and/or analysed during the current study are available from the corresponding author on reasonable request.
