# Supplementary material for: Challenges constraining availability and affordability of insulin in Bengaluru region (Karnataka, India): evidence from a mixed-methods study
Source: J Pharm Policy Pract. 2019 Oct 17;12:31. doi: 10.1186/s40545-019-0190-1 (PMC6796433; doi:10.1186/s40545-019-0190-1)
Supplement: Supplementary file 1 — Supplementary details on study methods, data analyses, and insulin availability and affordability. (PDF 457 kb) [file 40545_2019_190_MOESM1_ESM.pdf]

Satheesh et al. *Journal of Pharmaceutical Policy and Practice*
https://doi.org/10.1186/s40545-019-0190-1

Additional file 1

Bengaluru consists of five administrative zones, namely Bengaluru North, Bengaluru South, Bengaluru East, Bengaluru West and Bengaluru Central. In addition, Bengaluru further expands to peripheral towns. We conducted a representative facility survey – in four randomly selected zones and an additional peripheral town. In each of the five survey zones, we selected one public-sector hospital as the "survey anchor" and one private-sector hospital (i.e. total 10 hospitals). We also obtained insulin price data from the websites of various online pharmacies supplying insulin in Bengaluru.

Table S1a: List of the survey zones and public-sector 'survey anchor' hospital facilities

S. No.	Survey Zone	Public-sector 'anchor' hospital facilities (location)	Bed-count
1a.1	Bengaluru Central	Victoria Hospital* (near City Market)	1,000
1a.2	Bengaluru Central[±]	Bowring and Lady Curzon Hospital* (Shivajinagar)	886
1a.3	Bengaluru South	Jayadeva Institute of Cardiovascular Sciences & Research* (Jayanagar)	1,150
1a.4	Peripheral Town	Anekal Taluk Hospital** (Anekal)	100
1a.5	Bengaluru East	State government health scheme (near Lion's Airport City Hospital**)	35

*Tertiary care hospital; ** Secondary care hospital

[±]As 2 of the 3 major public sector hospitals in Bengaluru are located in Bengaluru Central, we selected two anchors from the same survey zone. Bengaluru West has neither tertiary nor secondary care facilities in the public sector.

Table S1b: List of survey districts and private sector hospital facilities

S.no.	Survey Zone	Private-sector hospital facilities	Bed-count
1b.1	Peripheral Town	Narayana Hrudayalaya* (Chandapura)	1,000
1b.2	Bengaluru West	Rajarajeshwari Medical College Hospital* (Kengeri)	1,300
1b.3	Bengaluru East	Sri Satya Sai General Hospital* (Whitefield)	330
1b.4	Bengaluru South	Sagar Apollo Hospital* (Jayanagar)	250
1b.5	Bengaluru Central	Mallya Hospital* (Vittal Mallya Road)	220

Note: Private sector hospitals do not serve as survey anchors, and were surveyed only due to its vast dominance over the public sector healthcare in Bengaluru district.

Table S1c: List and web links of the surveyed online private-sector pharmacies

1c.1	1mg	https://www.1mg.com/
1c.2	Apollo	https://www.apollopharmacy.in/
1c.3	MedPlus	https://www.medplusmart.com/
1c.4	NetMeds	https://www.netmeds.com/

Table S2: Occurrence of insulin (any brand or company) in the private-sector retail pharmacies in Bengaluru region, India

Insulin type	Molecule description	Strength	Brand (company)	Occurrence % (n)
**Human insulin**	Soluble insulin	40 IU/ml	Insugen R (Biocon)	44.82% (13)
			H.Actrapid (Novo Nordisk)	82.75% (24)
			Huminsulin R (Eli Lilly)	6.89% (2)
		100 IU/ml	Wosulin R (Wockhardt)	10.34% (3)
			H. Actrapid (Novo Nordisk)	31.03% (9)
			Insugen R (Biocon)	10.34% (3)
	Isophane insulin	40 IU/ml	H. Insulatard (Novo Nordisk)	20.68% (6)
			Insugen N (Biocon)	51.72% (15)
			Huminsulin N (Eli Lilly)	17.24% (5)
		100 IU/ml	Insulatard (Novo Nordisk)	17.24% (5)
			Insugen N (Biocon)	10.34% (3)
	Biphasic isophane insulin: 30% soluble insulin 70% isophane insulin	40 IU/ml	Insugen (Biocon)	44.82% (13)
			H. Mixtard (Novo Nordisk)	93.10% (27)
			Lupisulin M30 (Lupin)	34.48% (10)
			Insucare M30 (Ranbaxy)	13.79% (4)
			Huminsulin (Eli Lilly)	58.62% (17)
		100 IU/ml	Insugen (Biocon)	44.82% (13)
			Huminsulin (Eli Lilly)	27.58% (8)
			Mixtard (Novo Nordisk)	51.72% (15)
	Biphasic isophane insulin: 50% soluble insulin 50% isophane insulin	40 IU/ml	Insugen 50/50 (Biocon)	13.79% (4)
			H. Mixtard 50 (Novo Nordisk)	6.89% (2)
		100 IU/ml	Mixtard 50 (Novo Nordisk)	3.44% (1)
**Analogue insulin**	Insulin aspart	100 IU/ml	Novorapid (Novo Nordisk)	34.48% (10)
	Biphasic insulin aspart: 30% insulin aspart & 70% protamine crystallized aspart	100 IU/ml	Novomix 30 (Novo Nordisk)	37.93% (11)
	Insulin glargine	100 IU/ml	Basalog (Biocon)	27.58% (8)
			Lantus (Sanofi)	58.62% (17)
	Insulin glulisine	100 IU/ml	-	-
	Insulin lispro	100 IU/ml	Humalog Plain (Eli Lilly)	6.89% (2)
	Insulin lispro biphasic injection: 25% insulin lispro 75% lispro protamine	100 IU/ml	Humalog 25 (Eli Lilly)	10.34% (3)
			Eglucent Mix 25 (Lupin)	3.44% (1)
	Insulin lispro biphasic injection: 50% insulin lispro 50% lispro protamine	100 IU/ml	Humalog 50 Kwikpen (Eli Lilly)	20.68% (6)
	Insulin detemir	100 IU/ml	Levemir (Novo Nordisk)	3.44% (1)

**Table S3: Occurrence of insulin (any brand or company) in the public-sector hospitals in Bengaluru region, India**				
**Insulin type**	**Molecule description**	**Strength**	**Brand (company)**	**Occurrence % (n)**
**Human insulin**	Soluble insulin	40 IU/ml	H. Actrapid (Novo Nordisk)	60% (3)
			Insugen R (Biocon)	20% (1)
			Humarap (Cadila Healthcare)	20% (1)
		100 IU/ml	H. Actrapid (Novo Nordisk)	20% (1)
	Isophane insulin	40 IU/ml	Insugen N (Biocon)	20% (1)
		100 IU/ml	-	-
	Biphasic isophane insulin: 30% soluble insulin 70% isophane insulin	40 IU/ml	Insugen (Biocon)	60% (3)
			H.Mixtard (Novo Nordisk)	60% (3)
			Humstard (Cadila Healthcare)	20% (1)
			Lupisulin M30 (Lupin)	20% (1)
		100 IU/ml	H. Mixtard (Novo Nordisk)	60% (3)
			Insugen (Biocon)	20% (1)
			Huminsulin (Eli Lilly)	20% (1)
	Biphasic isophane insulin: 50% soluble insulin 50% isophane insulin	40 IU/ml	Huminsulin 50/50 (Eli Lilly)	20% (1)
		100 IU/ml	-	-
**Analogue insulin**	Insulin aspart	100 IU/ml	Novorapid (Novo Nordisk)	20% (1)
	Biphasic insulin aspart: 30% insulin aspart & 70% protamine crystallized aspart	100 IU/ml	Novomix 30 (Novo Nordisk)	40% (2)
	Insulin glargine	100 IU/ml	Basalog (Biocon)	40% (2)
			Lantus Solostar (Sanofi)	20% (1)
	Insulin glulisine	100 IU/ml	-	-
	Insulin lispro	100 IU/ml	-	
	Insulin lispro biphasic injection: 25% insulin lispro 75% lispro protamine	100 IU/ml	-	-
	Insulin lispro biphasic injection: 50% insulin lispro 50% lispro protamine	100 IU/ml	Humalog 50 Kwikpen (Eli Lilly)	20% (1)
	Insulin detemir	100 IU/ml	-	-

Table S4 : Occurrence of insulin (any brand or company) in the private-sector hospitals in Bengaluru region, India

Insulin type	Molecule description	Strength	Brand (company)	Occurrence % (n)
**Human insulin**	Soluble insulin	40 IU/ml	H. Actrapid (Novo Nordisk)	40% (2)
			Insugen R (Biocon)	60% (3)
			Huminsulin R (Eli Lilly)	40% (2)
		100 IU/ml	H. Actrapid (Novo Nordisk)	80% (4)
	Isophane insulin	40 IU/ml	Insugen N (Biocon)	80% (4)
			Huminsulin N (Eli Lilly)	60% (3)
			Insulatard (Novo Nordisk)	20% (1)
		100 IU/ml	Insulatard (Novo Nordisk)	20% (1)
	Biphasic isophane insulin: 30% soluble insulin 70% isophane insulin	40 IU/ml	Insugen (Biocon)	40% (2)
			Huminsulin (Eli Lilly)	40% (2)
			H.Mixtard (Novo Nordisk)	80% (4)
			H.Monotard (Novo Nordisk)	20% (1)
			Wosulin (Wockhardt)	20% (1)
			Lupisulin M30 (Lupin)	20% (1)
		100 IU/ml	H. Mixtard (Novo Nordisk)	100% (5)
			Insugen (Biocon)	60% (3)
			Huminsulin (Eli Lilly)	20% (1)
	Biphasic isophane insulin: 50% soluble insulin 50% isophane insulin	40 IU/ml	Huminsulin 50/50 (Eli Lilly)	20% (1)
			Wosulin 50/50 (Wockhardt)	20% (1)
		100 IU/ml	-	-
**Analogue insulin**	Insulin aspart	100 IU/ml	Novorapid (Novo Nordisk)	60% (3)
	Biphasic insulin aspart: 30% insulin aspart & 70% protamine crystallized aspart	100 IU/ml	Novomix 30 (Novo Nordisk)	20% (1)
	Insulin glargine	100 IU/ml	Basalog (Biocon)	40% (2)
			Lantus Solostar (Sanofi)	60% (3)
	Insulin glulisine	100 IU/ml	-	-
	Insulin lispro	100 IU/ml	Humalog Plain (Eli Lilly)	20% (1)
	Insulin lispro biphasic injection: 25% insulin lispro 75% lispro protamine	100 IU/ml	-	-
	Insulin lispro biphasic injection: 50% insulin lispro 50% lispro protamine	100 IU/ml	Humalog 50 Kwikpen (Eli Lilly)	60% (3)
	Insulin detmir	100 IU/ml	Levemir (Novo Nordisk)	20% (1)
**Porcine insulin**	Neutral porcine solution	40IU/ml	Actrapid Mc (Novo Nordisk)	20% (1)
	Porcine zinc suspension	40 IU/ml	Zinulin (Nicholas Pharma)	20% (1)

Table S5: Comparison of availability of insulin products – Chain pharmacy Vs. Independent pharmacy

Insulin type	Description	Presentation	Private retail sector	
			Chain pharmacy (n=10)	Independent pharmacy (n=20)
**Human insulin**	Soluble insulin	Vial (40 IU/ml)*	90% (n=9)	80% (n=16)
		Vial (100 IU/ml)	70% (n=7)	5% (n=1)
		Pen (100 IU/ml)	40% (n=4)	5% (n=1)
	Isophane insulin	Vial (40 IU/ml)*	100% (n=10)	35% (n=7)
		Vial (100 IU/ml)	30% (n=3)	
		Pen (100 IU/ml)	40% (n=4)	5% (n=1)
	Biphasic isophane insulin: 30% soluble insulin 70% isophane insulin	Vial (40 IU/ml) *	100% (n=10)	90% (n=18)
		Vial (100 IU/ml)	70% (n=7)	55% (n=11)
		Pen (100 IU/ml)	40% (n=4)	10% (n=2)
		Cartridge (100 IU/ml)	60% (n=6)	10% (n=2)
	Biphasic isophane insulin: 50% soluble insulin 50% isophane insulin	Vial (40 IU/ml)	30% (n=3)	15% (n=3)
		Vial (100 IU/ml)	0	0
		Pen (100 IU/ml)	0	0
		Cartridge (100 IU/ml)	0	5% (n=1)
**Analogue insulin**	Insulin aspart	100 IU/ml	50% (n=5)	25% (n=5)
	Biphasic insulin aspart: 30/70	100 IU/ml	90% (n=9)	10% (n=2)

	Insulin glargine	100 IU/ml	40% (n=4)	10% (n=2)
		100 IU/ml	70% (n=7)	55% (n=11)
	Insulin glulisine	100 IU/ml	0	0
	Insulin detemir	100 IU/ml	0	5% (n=1)
	Insulin lispro	100 IU/ml	20% (n=2)	0
	Insulin lispro biphasic 25/75	100 IU/ml	10% (n=1)	15% (n=3)
	Insulin lispro biphasic 50/50	100 IU/ml	50% (n=5)	5 % (n=1)
**Mean availability of insulin listed on India's 2015 Essential Medicines List**			**96.7%**	**68.3%**

**Table S6: Affordability of insulin in Bengaluru region, India**				
**6a. Private sector (Retail pharmacies and hospital pharmacies)**				
	**Median cost / 100 IU; 10ml vial (all companies)**	**Monthly Per capita cost of insulin (~ 1.2 vials or 1200 IU)**	**Ratio (monthly per capita insulin cost / per capita monthly health spending)**	**Number of man-days (unskilled labour) to pay for monthly insulin supply for a T1DM Child**
Human Vial	323.5	388.2	0.91	1.4
Human Cartridge	796.6	955.92	2.26	3.45
Human pen	1170	1404	3.32	5.06
Analogue Vial	1168.8	1402.56	3.31	5.05
Analogue Cartridge	1794	2152.8	5.09	7.76
Analogue pen	2160	2592	6.12	9.34
Porcine Vial	**302.95**	**363.54**	**0.86**	**1.31**
**6b. Public sector hospital pharmacies**				
	**Median cost /100 IU 10ml vial (all companies)**	**Per capita cost of monthly insulin supply (~ 1.2 vials or 1200 IU)**	**Ratio (monthly per capita insulin cost compared to India's per capita monthly health spending)**	**Number of man-days (unskilled labour) to pay for monthly insulin supply for a T1DM Child**
Human Vial	321	385.2	0.91	1.39
Human Cartridge	881.6	1057.92	2.5	3.81
Human pen	1170	1404	3.32	5.06
Analogue Vial	1232.8	1479.36	3.49	5.33
Analogue Cartridge	1590	1908	4.51	6.87
Analogue pen	2136.65	2563.98	6.06	9.24
The prices (INR) of all surveyed insulin products with variable pack sizes (milliliters) and strength were adjusted to 10 ml 100 IU/ml pack for purpose of comparisons. Per capita monthly insulin need is calculated based on WHO daily defined dose of 40 IU/day (i.e. 1200 IU/month). The wage of a lowest paid unskilled				

worker in Bengaluru region during the time of survey was Rs. 8,322 per month (i.e. daily wage of Rs. 277.4) The 2013 annual per capita health expenditure (both public and private sectors combined) was USD 78.0 (Rs. 5075.85) i.e. monthly USD 6.5 (Rs. 422.99)

Wholesaler interview guide

- On what basis do you decide on what insulin products to stock/supply?
- In Bangalore market, we found that more pharmacies stocked insulin products by non-Indian companies (Novo, Lily, Sanofi) compared to those by Indian companies (Biocon, Wockhardt, Cadila). What do you think is the reason?
- We noticed that, compared to findings from a study in Delhi state, the private retail pharmacies in Bengaluru have relatively more insulin products by Indian companies such as Biocon and Cadilla. What might be the reason for such difference between Delhi and Bangalore? Please answer based on your best understanding.
- In our quantitative analyses, we observed that a high proportion of surveyed pharmacies stock insulin analogue products (such as glargine). Why so many pharmacies have analogues (and cartridge/pen devices) while the government's essential medicine list on include human insulin.
- Among all human insulin products, which product and company has higher demand? Is Actrapid (and Mixtard) one of the high sales volume product, if yes what could be the reasons?
- What are your views on the online pharmacies? We found that most of the insulin products are sold by online pharmacies and some even offer cheaper prices compared to the private retail pharmacies. Comments?
- Compared to private retail sector, the public (government hospitals) have more Indian insulin products than non-Indian products. What might be the reason? Your thoughts?
- Do patients ever buy insulin directly from wholesalers? If yes, please explain what could be the reasons?
- Can you provide us the wholesaler selling prices of insulin products that you supply (on basis of anonymity)?
